# Connectivity of the Brain in the Light of Chemogenetic Modulation of Neuronal Activity

**DOI:** 10.32607/actanaturae.11895

**Published:** 2023

**Authors:** N. N. Dygalo

**Affiliations:** Institute of Cytology and Genetics, Siberian Branch of Russian Academy of Sciences (IC&G SB RAS), Novosibirsk, 630090 Russian Federation

**Keywords:** brain connectivity, functional magnetic resonance imaging, chemogenetics, neuronal activity, behavior

## Abstract

Connectivity is the coordinated activity of the neuronal networks responsible for brain functions; it is detected based on functional magnetic resonance imaging signals that depend on the oxygen level in the blood (blood oxygen level-dependent (BOLD) signals) supplying the brain. The BOLD signal is only indirectly related to the underlying neuronal activity; therefore, it remains an open question whether connectivity and changes in it are only manifestations of normal and pathological states of the brain or they are, to some extent, the causes of these states. The creation of chemogenetic receptors activated by synthetic drugs (designer receptors exclusively activated by designer drugs, DREADDs), which, depending on the receptor type, either facilitate or, on the contrary, inhibit the neuronal response to received physiological stimuli, makes it possible to assess brain connectivity in the light of controlled neuronal activity. Evidence suggests that connectivity is based on neuronal activity and is a manifestation of connections between brain regions that integrate sensory, cognitive, and motor functions. Chemogenetic modulation of the activity of various groups and types of neurons changes the connectivity of the brain and its complex functions. Chemogenetics can be useful in reconfiguring the pathological mechanisms of nervous and mental diseases. The initiated integration, based on the whole-brain connectome from molecular-cellular, neuronal, and synaptic processes to higher nervous activity and behavior, has the potential to significantly increase the fundamental and applied value of this branch of neuroscience.

## INTRODUCTION

One of the main goals in neurobiology is to establish the relationship between the brain neuronal activity and the higher nervous system functions, including normal and pathological cognitive and psycho-emotional states. Recent experimental and clinical data demonstrate a significant contribution of not only neurotransmitter, neuroendocrine, neurotrophic, immunologic, molecular and genetic regulators [1, 2, 3, 4, 5, 6, 7, 8], but also interaction between brain structures (connectivity [9, 10, 11, 12, 13, 14, 15]), which is the coordinated activity of the neuronal networks responsible for one of the brain functions, to the mechanisms underlying brain functions. For instance, this is evidenced by the observed relations between the activity of neuronal networks and attention [16, 15], memory [11, 17], as well as oth er many behavioral and psychological manifestations. [18, 19, 20]. A direct or indirect effect a group of neurons has on another group is called effective activity

The increased interest in brain connectivity is due to the promise of intravital non-invasive registration of its manifestations using functional magnetic resonance imaging (fMRI). This tool makes it possible to receive blood oxygen level-dependent (BOLD) signals from the blood supplying the brain. The local blood oxygenation level and, thus, the intensity of the BOLD signal, are believed to correspond to the general neuronal activity in the structure. The correlation between low-frequency fluctuations in BOLD signals from distant brain regions at rest, as well as upon brain activation by either sensory stimulation or pharmacological load is considered a measure of the connectivity between the structures [12].

Despite an increase in the number of studies in this field of neuroscience, the question of whether connectivity and its disorders are either one of the manifestations of normal or pathological states of the brain, or the causes of these states, remains open. In order to answer this question, one should distinguish the causal relationships between these states and connectivity from random correlations between them. Experimental effects triggering a functional brain response (behavioral, vegetative, and other responses) and changes in the connectivity accompanying them are not sufficient to establish a causal relationship between them. Both induced responses and changes in connectivity can be independent manifestations of the state resulting from the exposure. Specific physiological interventions changing neuronal activity and, presumably, connectivity, which is based on this activity, are required. The majority of these effects are not applicable to humans. For this reason, experiments are conducted on animals [21].

Chemogenetic methods, which have only recently come to the fore [22], have significantly improved our ability to study brain functions. These methods make it possible to control the activity of specific neurons using synthetic receptors; e.g., receptors to guanine nucleotide-binding proteins (G) and ligand-gated ion channels. Among these, designer receptors exclusively activated by designer drugs (DREADD) seem promising. Leveraging these receptors either facilitates or, on the contrary, inhibits the neuronal response to the natural physiological stimuli received, depending on the receptor type. At the same time, they do not impose the activity of the effect external to the brain and neuron. Therefore, they make it possible to take the most objective look at the neuronal network organization of brain functions in the light of neuronal activity modulation. The description and systematization of the available data on this issue are the goal of this study.

## DESIGNER RECEPTORS EXCLUSIVELY ACTIVATED BY DESIGNER DRUGS

DREADD is the most popular chemogenetic approach used to study the regulation of various aspects of brain activity by neuronal networks in experimental animals [22, 23, 24, 25, 26, 27, 28]. In addition to solving fundamental science tasks, chemogenetics can potentially become useful for restructuring the pathological mechanisms of nervous and mental diseases and regulate them using designer drugs [29]. DREADDs interact with exogenous synthetic ligands, which are inert to natural body receptors. The activity of neurons expressing these synthetic receptors can either be enhanced or inhibited, depending on the type of the receptor used. DREADD variants and the features of their expression by the viral vectors and transgenes used in experiments for assessing their chemogenetic effects on brain connectivity are presented below.

Two DREADD variants based on the human muscarinic acetylcholine receptors (hM3D(Gq) and hM4D(Gi)) are widely used. To create DREADD-hM3D(Gq), which enhances the neuronal response to physiological stimuli upon interaction with the synthetic ligand clozapine N-oxide (CNO), a metabotropic type 3 acetylcholine receptor, coupled with the activating guanine nucleotide-binding protein Gq, was used. The functional effect of these receptors is considered to be due to an increase in neuronal sensitivity to the stimulation resulting from neuron membrane depolarization. DREADDhM4D( Gi) inhibiting activation of a cell with these receptors upon interaction with CNO was developed using a modified type 4 metabotropic acetylcholine receptor, coupled to the inhibitory guanine nucleotide- binding protein (Gi). Receptor hM4D(Gi) is considered to decrease neuronal excitation though cell hyperpolarization. In addition to acetylcholine receptors, the kappa-opioid receptor, coupled with the inhibitory guanine nucleotide-binding Gi (KORD), which inhibits cell activity upon interaction with its pharmacologically inert ligand salvinorin b, was used [30]. Chemogenetic inhibition/inactivation in experimental neurobehavioral neuroscience is often referred to as chemogenetic silencing. DREADDs enter the brain either as part of a transgene, whose expression in the structure of interest is achieved by crossing specific mouse lines [31], or as part of a vector, which is usually based on an adeno-associated virus [24, 25, 26, 27, 28]. Stereotaxic administration of the viral vector provides its localization in the brain structure of interest, while the promoter governing the expression ensures the presence of the DREADD in the desired type (or types) of cells.

DREADDs are well expressed in the central nervous system cells as part of viral vectors or transgenes and can be used for reversible activation and inhibition of target cells upon systemic administration of the ligands to these receptors or direct injection of these ligands into the brain. High expression level in the brain is provided by promoters nonselective to the neuron type, such as cytomegalovirus [32] and human synapsin (hSyn) promoters [31, 32, 33, 34, 35, 36]. The use of promoters that are active only in certain neurons, such as CaMKII, which is active in glutamatergic pyramidal neurons [31, 37], promoters specific to GABAergic interneurons synthesizing parvalbumin and expressing prodynorphin or proenkephalin [31], as well as serotonergic- [38], noradrenergic-specific, and dopamine-responding neurons [39, 40, 41], makes it possible to study the effect of a change in the activity of a certain neuron type and subtype on brain connectivity.

## CONNECTIVITY

Functional connectivity is determined based on the correlation between BOLD signals measured in different brain regions by functional magnetic resonance imaging (fMRI). The signal registered in the absence of external stimuli is considered resting-state fMRI (rs-fMRI). BOLD signals associated with task performance are called responses; e.g., in tactile fMRI used in limb stimulation [42]. Spatial maps showing the intrinsic functional network organization of the brain are obtained based on these signals [43].

Each network consists of groups of neurons that are located in different structures of the brain but respond in a coordinated way, with changes in their activity depending on the external and/or internal stimuli. For instance, the default mode network (DMN) is responsible for cogitation and self-reflection, which are independent of external stimuli in a healthy brain. Inhibition of its activity improves the performance of tasks requiring attention [9]. The DMN core includes the posterior medial and the parietal brain cortices, as well as separate zones of the temporal and frontal lobes [44]. The salience network (SN) fosters attention to important events; it perceives and responds to signals related to homeostasis [45]. The main SN structures are anterior insula, the anterior cingulate cortex, and ventral striatum. The central executive network (CEN) operates with working memory information; it is responsible for making decisions in goal-informed behavior. Its centers are the dorsolateral prefrontal and the posterior lateral parietal cortices. These and other largescale networks that were first identified in humans [46] demonstrate pronounced homology with similar networks in monkeys and rodents [47, 48].

The activity of the functional connectivity networks assessed by rs-fMRI correlates well with cognitive abilities and behavior [49], as well as changes in the brain excitation level [10, 50]. It is of practical importance that rs-fMRI signals in patients with mental [13, 20, 51, 52] and neurodegenerative [53] diseases clearly differ in functional connectivity from those of a healthy human brain. Despite the fact that they make it possible to use rs-fMRI for disease diagnosis [54], the results related to these differences are usually difficult to interpret, since the BOLD signal is only weakly and indirectly related to the underlying neuronal activity.

For this reason, the right approaches to identifying any possible relation between regional functional connectivity and direct indicators of neuron activation, neurotransmitter release, and metabolism in brain cells are currently being sought. In order to do this, it is necessary to induce changes in neuronal activity and register either the rs-fMRI or positronemission tomography (PET) signals. The latter method is attempted much less frequently than rs-fMRI, since it requires the use of positron-emitting radioisotopes, such as 18F-fluorodeoxyglucose (18-FDG) [55]. Transcranial magnetic stimulation used in humans indeed affects DMN connectivity [56]. However, changes in the activity of neurons beyond the scope of its normal patterns affect network structure and function. In this regard, only modulation within the natural range can be used to assess the effect of neuronal activity on the connectivity [57]. The chemogenetic approach is the most suitable among the others (pharmacological, electrophysiological, magnetic field exposure- based, and optogenetic methods) when seeking to alter neuronal activity. This approach mainly facilitates or, on the contrary, inhibits the neuronal response to natural physiological stimuli. Some variants of these methods were given specific names by the authors. For instance, the method including the DREADD approach and 18F-fluorodeoxyglucose imaging by PET is called DREADD-assisted metabolic mapping [55]. The combination of DREADD and rsfMRI to study changes in spontaneous neuronal activity is called chemo-fMRI [38].

It should be noted that activation of nodal neurons affects even distant regions of the brain. For instance, chemogenetic stimulation of neurons expressing the D1 dopamine receptor in the dorsal striatum of only one hemisphere activates electrophysiological responses in the medial thalamus, nucleus accumbens, and both hemisphere cortices in mice [41]. Inhibition of neuronal activity in nodal regions can also elicit a spiking nature of propagation that goes beyond the connections with the node and affects other neuronal networks [35].

The bulk of the experimental data available to date, which are to be discussed below, indicate a change in the connectivity upon chemogenetic modulation of the activity of different groups and types of brain neurons.

## MODULATION OF NEURONAL ACTIVITY NON-SPECIFIC TO NEURONAL TYPE

Currently, a number of laboratories use chemogenetic modulation of neuronal activity non-selective to the neuron type, in combination with fMRI [34, 35, 36, 37, 38, 39, 58], to study the responses of the neuronal network connectivity in laboratory animals.

The mammalian brain is a complex system, and a change in neuronal activity even in one region can lead to large-scale effects on its many functions. For instance, chemogenetic inactivation of all types of neurons expressed under the hSyn DREADD–hM4D promoter, which inhibits the activity of the neurons in the amygdala, a subcortical region with broad connections in the cortex, disrupted the amygdalocortical fMRI connectivity and the distribution of corticocortical connections between functional brain networks in rhesus macaques [33]. A higher number of DREADDtransfected cells in the amygdala was associated with a more pronounced disruption of the functional connectivity between this structure and monosynaptically connected, as well as non-directly connected, brain regions. The combination of communication contacts through the monosynaptic and polysynaptic pathways explains to a large extent the correlational structure of endogenous brain activity and many of the changes in it resulting from a decrease in amygdala activity. These results indicate a structural basis for neuronal activity and a possible relation between neuropathology and neurophysiological changes in regions distant from the presumptive focus [33].

The DMN is the main network in the mammalian brain. However, the functional role of the nodes in this network and the mechanisms underlying the connection between its structure and the behavior it regulates remain unclear. To gather information on these issues, chemogenetic inactivation of the key DMN node, namely the dorsal anterior cingulate cortex, was used, in combination with rs-fMRI and behavioral testing in awake rats [34]. The authors called this method the hemo-rsfMRI-behavior approach. Inhibition of the activity of the dorsal anterior cingulate cortex using DREADD-hM4Di, expressed under the hSyn promoter, significantly deactivated the neurons in the prelimbic and dorsal cortices of the middle cingulate gyrus and induced multidirectional changes in connectivity between DMN nodes. The changes in the connectivity correlated with the animal’s behavior: anxiety and motor activity in the home cage were noted. The results indicate that DMN activity in both rodents and humans is aligned with behavior [34].

Chemogenetic activation of the mesolimbic and mesocortical pathways, which are projections of the ventral tegmental area to the nucleus accumbens and medial prefrontal cortex (PFC), respectively, induced BOLD responses not only in DREADD-expressing regions, but also in neuronal networks distant from the sites of the chemogenetic vector injections not expressing DREADD [36]. The neurochemical nature of these pathways is apparently heterogeneous. This conclusion is based on the fact that the hSyn promoter, which is nonselective to the neuron type, was used for DREADD-hM3D(Gq) expression, while projections from the ventral tegmental region to the nucleus accumbens release dopamine, glutamate, GABA, the brain-derived neurotrophic factor, and other signaling molecules [59]. The duration of the brain activity corresponded to the onset of the behavioral response: motor hyperactivity in animals with chemogenetically induced mesolimbic pathway. This activation specifically increased neuronal activity, while functional connectivity measured by rs-FMRI remained stable. Positive and negative BOLD signals clearly showed simultaneous activation of the ventral pallidum and deactivation of the pars reticulata of the substantia nigra, respectively, by demonstrating coordinated reversely directed changes in the activity of different areas of the neuronal network after the stimulation of specific midbrain projection neurons [36]. It should be noted that, in contrast to Roelofs et al. [36], who noted the stability of the functional connectivity in chemogenetic activation of subcortical pathways, other authors mentioned in the present review observed changes in functional connectivity upon chemogenetic modulation of the activity of different neurons in various brain structures.

Acute chemogenetic inhibition of PFC neurons by DREADD-hM4D(Gi) under the nonselective hSyn promoter enhanced fMRI connectivity between this region and its direct thalamocortical targets. PFC inhibition increased the power of low-frequency oscillations by reducing the discharge activity of neurons, which was unrelated in phase to slow the rhythms. This led to an increase in coherence between the slow and *δ*-bands of the electroencephalogram rhythms between the regions demonstrating fMRI overconnectivity. Apparently, cortex inactivation can increase fMRI connectivity through the enhancement of slow oscillatory processes [31].

Simultaneous chemogenetic reactivation of a set of many of the neuron ensembles involved in the formation of the memory engrams responsible for threat processing and associated with increased expression of the early response gene *c-fos *in these conditions, which are functionally associated, in particular, with hippocampus and amygdala neurons, yielded a more effective behavioral engram, compared to the reactivation of only one ensemble, and reproduced the fear factor caused by the threatening situation more fully. These results show that connectivity of distant structures is a natural occurrence in the implementation of complex brain functions [60].

Chemogenetic inhibition of connectivity as relates to the orbitofrontal cortex and the rostromedial caudate nucleus in rhesus macaques through a contralateral expression of the inhibitory DREADD hM4Di in these brain structures disrupted the capacity to adequately capture the food reward value [32]. In these experiments, DREADD expression was enabled by the cytomegalovirus promoter and the disrupted connectivity could not have been due to a decrease in the activity of any particular type of neurons or glial cells [61]. Nevertheless, it is clear that connectivity in the orbitofrontal cortex and rostromedial caudate nucleus is likely crucial in the formation of motivated behavior based on the integration of external stimuli with the internal drive of monkeys [32].

In addition to nonselective modulation of neuronal activity, changes in the activity of any particular type of neurons also affected connectivity and, apparently, the manifestation of the higher brain functions controled by it.

## MODULATION OF NEURONAL ACTIVITY IN MONOAMINERGIC NEUROTRANSMISSION

Chemogenetic-induced tonic activation of noradrenergic norepinephrine (NE) neurons in the locus coeruleus (LC) in the pontine region of the mouse brain led to a reduction in its blood supply and glucose uptake because of these neurons. What is more, it also increased the synchronous low-frequency fMRI activity in the frontal cortex of the DMN, which is significantly distant from the LC. LC-NE activation induced NE release, enhanced neuronal calcium signals, and decreased blood supply into the anterior cingulate cortex. LC-NE stimulation also enhanced functional connectivity in the frontal DMN and, apparently, boosted the behavior associated with this brain network [40]. LC activation in humans is associated with a shift in connectivity amongst the brain networks in favor of processing of the most relevant information [62]. A possible causal relationship within this association was analyzed in mice by using chemogenetic activation of LC coupled with rs-fMRI [39]. This approach is called chemo-connectomics. LC activation was found to rapidly interrupt current behavior and significantly increase brain-wide connectivity, with the most pronounced effects being noted in the salience and amygdala networks. Changes in functional connectivity correlated with the levels of the alpha-1- and beta-1-adrenergic receptor transcripts in the brain, while functional network connectivity correlated with NE metabolism within the brain structures. It is likely that these changes in large-scale network connectivity affect the optimization of neuronal information processing, which is significant in increasing vigilance and detecting threats [39].

Chemogenetic activation of neurons expressing dopamine D1 receptors in the mouse left dorsal striatum increased the fractional amplitude of low-frequency fluctuations (fALFF) in the medial thalamus, nucleus accumbens, and the cortexes of both hemispheres. In addition, gamma-band local field potentials were increased in the stimulated striatum and the cortices of both hemispheres [41].

Serotonin-producing neurons abundantly innervate brain regions through their extended projections [63]. The use of chemo-fMRI to identify any possible effect of serotonergic neurotransmission on regional and global functional activity showed that endogenous stimulation of serotonin-producing neurons did not affect global brain activity but caused regional activation of a series of primary target regions encompassing the cortico-hippocampal and ventral striatal areas. At the same time, the pharmacological increase in serotonin levels led to widespread fMRI deactivation in the brain, which probably is an indication of a combined contribution of central and perivascular constrictive effects. These results identify the main functional targets of endogenous serotonergic stimulation and suggest a possible causal relationship between serotonergic neuron activation and regional fMRI signals [38].

## MODULATION OF THE ACTIVITY OF PYRAMIDAL NEURONS AND INTERNEURONS

Chemogenetic stimulation of the bed nucleus of the stria terminalis expressing the vesicular γ-aminobutyric acid (GABA) transporter using DREADD-hM3Dq prompted anxiety-like behavior and resulted in long-term depression in glutamatergic neurotransmission, indicating changes in synaptic plasticity. Metabolic mapping of whole-brain activity after such exposure revealed enhanced activity within the ventral midbrain structures, including the ventral tegmental area, and hindbrain structures such as the LC and the parabrachial nucleus. The activity of these brain nuclei is associated with anxiety-like behavior. The use of microfluidics profiling of the receptor system of individual neurons in the bed nucleus of the stria terminalis expressing the vesicular GABA transporter showed that stimulation of the Gq-coupled type 2c serotonin receptor is responsible for anxietylike behavior [64].

DREADD modulation combined with 18F-FDGPET imaging called DREADD-assisted metabolic mapping (DREAMM) made it possible to create whole-brain metabolic maps of animals under conditions of free behavior [65]. This method was used to demonstrate the association of various corticolimbic networks with specific manifestations during inhibition of the activity of prodynorphin- and proenkephalin- expressing inhibitory GABAergic medium spiny neurons in the nucleus accumbens shell [65], which are associated with neuropsychiatric disorders.

Decreased activity of glutamatergic neurons in the right anterior cingulate cortex in mice due to the effect of the inhibitory kappa-opioid receptor DREADD (KORD) expressed under the CaMKII promoter resulted in a reduced fMRI BOLD signal in this brain region and increased the fMRI BOLD signal in the brain regions of both hemispheres associated with the anterior cingulate cortex. Changes in neuronal activity were observed in functional networks, including connections with the sensory cortex, thalamus, basolateral amygdala, and globus pallidus (s. pallidum). These regions are responsible for attention, working memory, fear, and reward, respectively. This modulation of neuronal activity was accompanied by a decrease in intra- and interhemispheric functional connectivity [58].

Chemogenetic excitation of the main glutamatergic pyramidal neurons expressing activating DREADDhM3D( Gq) under the CaMKII promoter and inhibition of parvalbumin-expressing GABAergic interneurons in the PFC weakened the connection between the latter and DMN cortical projections. Both types of exposure increased the local excitation rate and shifted the local field potential (LFP) power towards higher frequencies, while effectively reversing the electrophysiological effects of the inhibitory DREADD-hM4D(Gi) expressed in the cortex under the nonselective hSyn promoter. Stimulation of pyramidal neurons suppressed slow- and deltaband LFP activity more effectively than interneuron inhibition. The functional overconnectivity observed in these experiments is assumed to be due to both an increased excitation-to-inhibition ratio in the PFC and a nonspecific functional decrease in the activity of GABAergic neurons [31].

Chemogenetic activation of the glutamatergic neurons of the paraventricular hypothalamic nucleus expressing the rAVV-CaMKIIα-hM3Dq-mCherry vector increased BOLD signals measured by chemo-fMRI and functional connectivity between paraventricular and olfactory nuclei, the cingulate cortex, the paraventricular thalamic nucleus, the periaqueductal gray nucleus, and the hippocampus after CNO administration to rats [66].

## NEURONAL ACTIVITY MODULATES BEHAVIOR THROUGH CONNECTIVITY

The results of recent experiments similar to the ones described above allow one to consider specific chemogenetically induced changes in connectivity not only as concomitant signs of higher nervous activity dysfunction, but also as a likely cause of the disorder. For instance, an association between corticolimbic networks and specific behavior manifestations was found upon inhibition of the activity of inhibitory GABAergic medium spiny neurons. Levorotatory behavior was increased in prodynorphin-expressing neurons, and dextrorotatory behavior was enhanced in proenkephalin-expressing neurons. Inhibition of neuronal activity in awake state and under anesthesia changed the activity of different neuronal networks [65]. Chemogenetic inactivation of the interaction between the orbitofrontal and rhinal cortices using hM- 4Di-DREADD reduced the ability of monkeys to discriminate among expected rewards in a behavioral experiment [67]. Chemogenetic impairment in the connectivity of the orbitofrontal cortex and the rostromedial region of the caudate nucleus changed the formation of motivated behavior due to a combination of external stimuli with the internal drive of monkeys [32]. Inhibition of activity in the dorsal anterior cingulate cortex using DREADD-hM4D(Gi) expressed under hSyn significantly decreased the activity of the neurons in the prelimbic and dorsal cortices of the middle cingulate gyrus and induced multidirectional changes in the connectivity between DMN nodes. These changes correlated with animal behavior: anxiety and motor activity in the home cage were noted. It is apparent that DMN activity in both rodents and humans is coordinated with behavior manifestations [34]. Chemogenetic activation of LC-NE induced NE release, enhanced neuronal calcium signals, and decreased blood supply to the anterior cingulate cortex. LC-NE activation also enhanced functional connectivity in the frontal DMN and, as a consequence, promoted a behavior response associated with this brain network [40].

Both contralateral and bilateral, but not ipsilateral, chemogenetic inactivation of predominantly glutamatergic neurons in two structures (dorsal hippocampus and PFC) impaired learning in rats in the W-maze. These results indicate that the connectivity of the dorsal hippocampus and PFC plays a key role in spatial learning and memory [68]. The combined use of chemogenetic inactivation of the activity of the primary somatosensory cortex using tactile fMRI revealed a link between neuronal activity, connectivity, and behavior in macaques. Focal chemogenetic silencing of the functionally identified hand region in the somatosensory cortex impaired grasping. The same inhibition also attenuated the fMRI signal induced by hand stimulation both at the local silencing site and anatomically and/or functionally connected downstream network underlying the induced grasping behavior disorder. In addition, inhibition of the hand region unexpectedly disinhibited foot representation, with concomitant behavioral hypersensitization [42].

## CONCLUSION

Connectivity is the manifestation of natural connections between brain regions that selectively integrate sensory, cognitive, and motor activation. These connections are rooted in brain evolution [69], and their individual features take shape during ontogeny [70]. The genetic component substantially contributes to the formation of individual connectome features. The majority of the 19 various neuropsychiatric and idiopathic conditions studied in more than 30,000 individuals had specific connectome profiles that correlated with the genomic and transcriptomic features of these conditions [71]. Genes play an important role in the formation of functionally important and metabolically costly interactions between the nodal regions of the connectomes. These regions share transcriptional activity patterns owing to the similarity of their metabolic and cytoarchitectonic features. The genes involved in the formation and maintenance of synapses and axons are important for establishing connections between different brain regions; in particular, the transcriptome features of the nodal centers of neural networks are determined by the metabolic needs of these centers [72, 73]. It should be noted that DREADD activation alters gene expression. For instance, chemogenetic activation of the glutamatergic neurons of the superior colliculus significantly changed the transcriptome of this structure towards the predominance of neurotrophic processes [74]. Thirteen defects in nervous system development, neurological and mental disorders, whose predictors are molecular genetic and biochemical disorders, were found to be associated with the structural and anatomical patterns of cortical anomalies affecting the main brain network architecture; this indicates a likely mutual enhancement of the negative contributions of local molecular and global connectome mechanisms to the pathology [75].

Many studies and reviews have discussed the variability of gene expression patterns in the brain in one psycho-emotional state, up to almost complete discrepancy between different mouse lines [76]. Therefore, one of the ways to clarify the structure and function of the mechanisms underlying these conditions may be to analyze the brain parameters that are more closely related to psycho-emotional regulation, such as connectivity, which is also regulated by gene expression [8]. The results available to date, including the ones discussed in the current review, provide sufficient evidence of this.

**Fig. 1:**
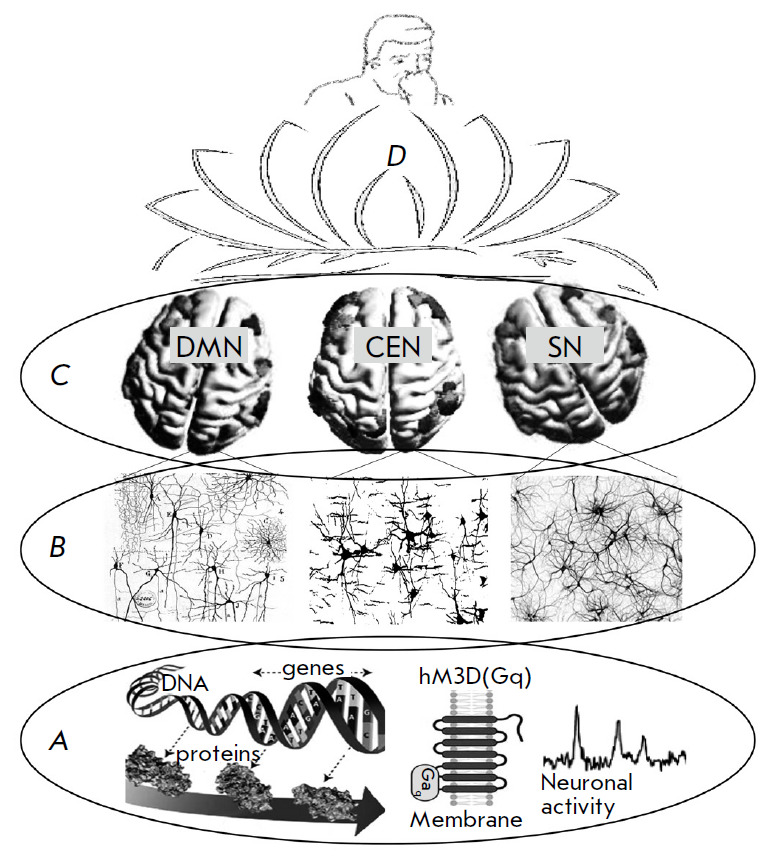
Schematic presentation of the multilevel organization of brain functions in mammals. (*A*) – molecular and cellular processes, genes, proteins, cell membrane with proteins (as an example, the chemogenetic activating hM3D (Gq) receptor is presented schematically), and electrophysiological activity of the neuron. (*B*) – neuronal network ensembles of neurons interconnected through contacts, which form the basis of the structural and functional organization of the brain. (*C*) – large-scale brain networks, each of which emerged during evolution based on neuronal ensembles, which are presented schematically in (*B*), for predominant performance of certain adaptive functions by each of them. Pictures of the apical surface of the human brain show three large-scale networks: the default mode network (DMN), the central executive network (CEN), and the salience network (SN). The most important sections of each of the networks are shaded. Apparently, the interactions of DMN, CEN, SN, as well as a number of other large-scale brain networks, ensure cognitive and behavioral manifestations in an individual (*D*)

In addition, identification of the biological meaning of the connectome requires not only its analysis data, but also the results of studies in related science fields such as anatomy, physiology, molecular genetics, and behavior analysis. Information regarding intracellular and cell properties, synapse plasticity, and the effects of neuromodulators on cells and synapses is of the utmost importance. Such data will make it easier to map out the entire pathway of connnectivity, from molecular and cellular, neuronal and synaptic processes to higher nervous activity and behavior based on the connectome (*Fig. 1*).

## Abbreviations

BOLD: blood oxygen level-dependent fMRI signal
CaMKII: calcium-calmodulin-dependent protein kinase II
CEN: central executive network
CNO: clozapine N-oxide
DREADD: designer receptors exclusively activated by designer drugs
DMN: default mode network
GABA: gamma-aminobutyric acid
Gq: activating guanine nucleotide-binding protein
fMRI: functional magnetic resonance imaging
rs-fMRI: functional magnetic resonance imaging at rest
hM3D(Gq): activating chemogenetic receptor
hM4D(Gi): inhibitory chemogenetic receptor
hSyn: human synapsin promoter
KORD: inhibitory chemogenetic receptor
LC: locus coeruleus
mCherry: red fluorescent protein
NE: norepinephrine
PFC: prefrontal cortex
rAVV: recombinant adeno-associated virus
SN: salience network.
